# Dupilumab probably reduces transepidermal water loss but does not increase stratum corneum hydration in atopic dermatitis

**DOI:** 10.1111/1346-8138.15638

**Published:** 2020-10-09

**Authors:** Takuya Furuhashi, Takao Oda, Kan Torii, Emi Nishida, Akimichi Morita

**Affiliations:** ^1^ Department of Dermatology Kasugai Municipal Hospital Kasugai Japan; ^2^ Department of Geriatric and Environmental Dermatology Nagoya City University Graduate School of Medical Sciences Nagoya Japan

Dear Editor,

Atopic dermatitis (AD) is a common and pruritic inflammatory skin disease, which has been widely treated by dupilumab.^1^ It contributes to the treatment of adolescent AD patients with unmet high medical needs^2^ and studies on the efficacy and safety in children as young as 6 years of age are in progress.^3^ However, it is not yet known whether dupilumab restores skin barrier function in AD patients. Therefore, we investigated the extent to which skin barrier function was restored following treatment with dupilumab, using transepidermal water loss (TEWL) and stratum corneum hydration (SCH). A total of seven patients (aged 40.4 ± 15.8 years) and seven age‐matched healthy volunteers (aged 35.4 ± 11.7 years) were enrolled in this study. Patients continued to use the same moisturizers and topical treatments that they had used before the study. The study was approved by the ethics committee of Kasugai Municipal Hospital, Kasugai, Japan. TEWL was measured by a VapoMeter^®^ (Delfin Technologies, Kuopio, Finland) with a closed unventilated chamber system. SCH was measured with a MoistureMeter SC^®^ (Delfin Technologies). Statistical analyses were performed using GraphPad Prism software version 7 (GraphPad, San Diego, CA, USA).

Although TEWL tended to be high on the forehead (controls vs patients, 22.7 ± 6.9 versus 40.7 ± 30.7) and cheek (20.2 ± 4.7 vs 43.5 ± 55.5), surprisingly none of the patients had high TEWL on their neck and arm (back of the neck, 20.0 ± 6.8 vs 20.9 ± 6.5; upper inner arm, 11.5 ± 2.3 vs 10.8 ± 3.3; forearm anterior, 11.7 ± 4.1 vs 10.7 ± 4.3; and forearm dorsum, 10.5 ± 1.6 vs 8.9 ± 1.8) (Fig. 1a). Furthermore, SCH was also significantly lower on their neck and arm (back of the neck, 52.6 ± 9.4 vs 31.6 ± 23.0; upper inner arm, 36.4 ± 18.4 vs 17.0 ± 7.5; forearm anterior, 36.2 ± 13.1 versus 17.4 ± 8.6; and forearm dorsum, 32.2 ± 18.9 vs 14.4 ± 5.3) (Fig. 1b). Following treatment with dupilumab, TEWL in the lesions decreased quickly (Fig. 1c), while SCH was not increased over half a year (Fig. 1d). Next, we investigated the changes in TEWL and SCH among six sites including non‐lesional. The SCH of the forehead and neck was increased temporarily, but returned to the baseline after approximately 14 weeks. We considered that dupilumab did not continue the beneficial effects for dry skin conditions (Fig. 1e,f). It may be difficult to express all skin barrier functions using TEWL and SCH measured with these devices, and a more precise instrument such as Raman spectroscopy may improve the accuracy of skin barrier function measurement.^4^

**Figure 1:**
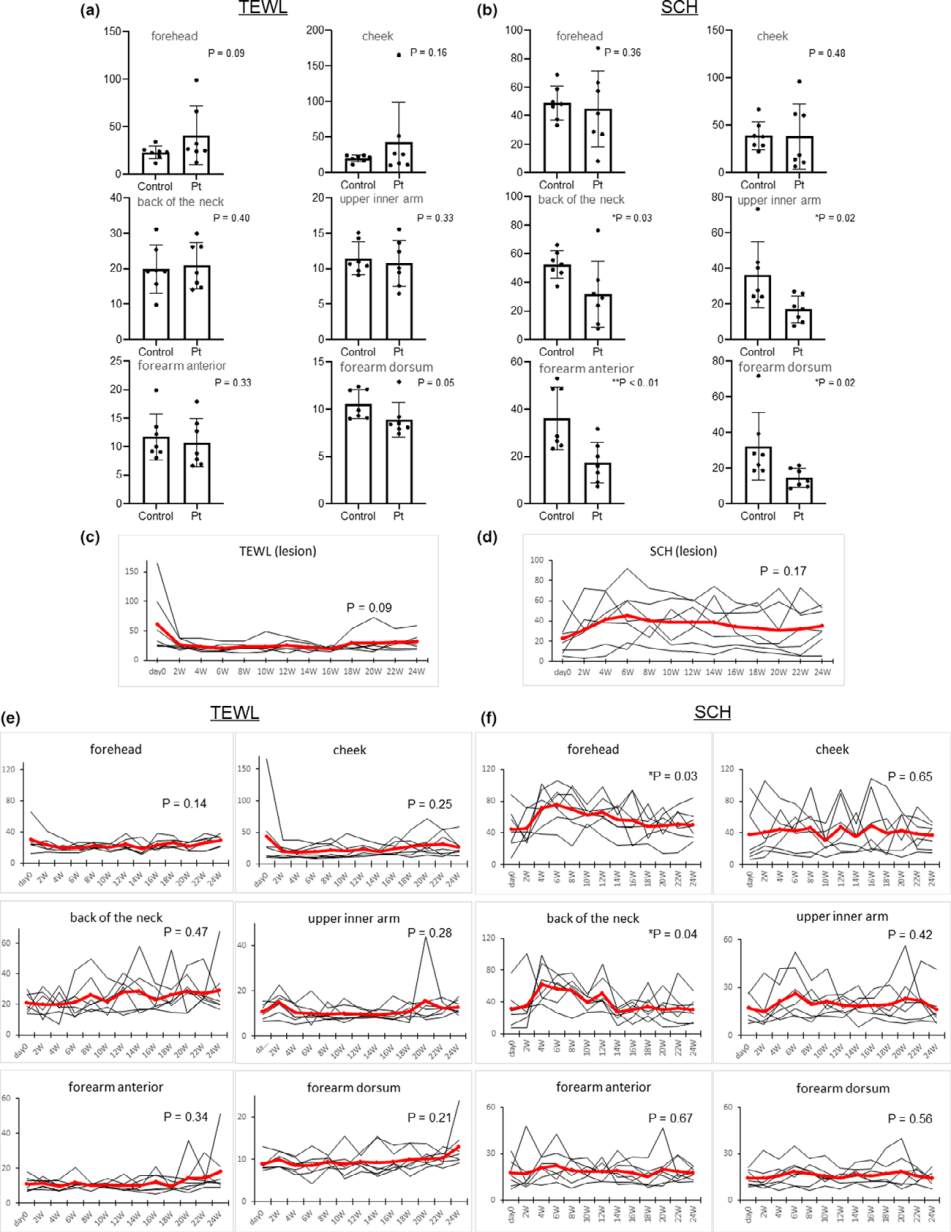
(a,b) Comparison of transepidermal water loss (TEWL) and stratum corneum hydration (SCH) between atopic dermatitis (AD) patients (Pt) and age‐matched controls (*n* = 7). Each site was measured three times, and the mean was used for analysis (Student’s *t‐*test). (c,d) Changes in TEWL and SCH levels in the lesions (hand, forehead, forearm anterior, forehead, cheek and forehead). Analyses were performed with two‐way anova and Dunnett’s test. (e) Changes in TEWL levels in six sites from the index date to 24 weeks. Analyses were performed with two‐way anova and Dunnett’s test. (f) Changes in SCH levels in six sites from the index date to 24 weeks.

In this study, the SCH of patients was significantly lower but not increased by dupilumab. Clinically, we also experience patients treated with dupilumab who have recovered from their dry skin condition. Because of the small sample size in this study, further studies are needed. SCH is very useful to objectively show dryness and maintain the patient’s motivation to moisturize. Although many new drugs for AD are expected to be developed in the future,^5^ it is important to consider how long remission can be maintained after the treatment is finished. Therefore, we conclude that it is necessary to continue skin care instruction during and after the treatment of dupilumab.

## Conflict of Interest

None declared.
